# Impact of In‐Office Dispensing Adoption by Urology Practices on Oral Specialty Drug Use in Advanced Prostate Cancer

**DOI:** 10.1002/cam4.71475

**Published:** 2026-01-11

**Authors:** Kassem S. Faraj, Mary Oerline, Samuel R. Kaufman, Christopher Dall, Arnav Srivastava, Xiu Liu, Megan E. V. Caram, Vahakn B. Shahinian, Brent K. Hollenbeck

**Affiliations:** ^1^ Dow Division of Health Services Research, Department of Urology University of Michigan Ann Arbor Michigan USA; ^2^ Department of Urology Massachusetts General Hospital Boston Massachusetts USA; ^3^ VA Health Services Research & Development, Center for Clinical Management Research, VA Ann Arbor Healthcare System Ann Arbor Michigan USA; ^4^ Division of Hematology/Oncology, Department of Internal Medicine University of Michigan Ann Arbor Michigan USA; ^5^ Division of Nephrology, Department of Internal Medicine University of Michigan Ann Arbor Michigan USA

**Keywords:** advanced prostate cancer, financial incentives, prostate cancer

## Abstract

**Introduction:**

A paradigm shift in advanced prostate cancer toward the use of oral specialty drugs has been accompanied by increased involvement of urologists. In fact, some urology groups can deliver these medications in the office, providing them directly to their patients.

**Methods:**

A retrospective cohort study was performed using a 20% national sample of men with advanced prostate cancer enrolled in Traditional Medicare and managed by independent urology groups between 2011 and 2019. Urology groups with and without in‐office dispensing were identified. Multivariable logistic regression was used to assess relationships between urology group characteristics and in‐office dispensing. A difference‐in‐differences design was used to measure the effect of in‐office dispensing on the use of oral specialty drugs within urology group markets compared to markets without dispensing.

**Results:**

Urology group characteristics associated with adoption of in‐office dispensing included large practice size (OR 2.9, 95% CI 1.2–6.5), increasing volume of men with advanced prostate cancer (OR 1.1 per 10‐patient increase, 95% CI 1.0–1.2), decreasing social vulnerability of the group's patient population (OR 0.81 per 0.1 unit increase, 95% CI 0.69–0.95), lower competition (OR 1.02 per 100 unit increase in the Herfindahl–Hirschman Index, 95% CI 1.0–1.1), and radiation vault ownership (OR 3.1, 95% CI 1.1–8.4). Compared with markets of urology groups that did not adopt in‐office dispensing, adopting markets experienced a significant increase in oral specialty drug prescriptions (adjusted difference‐in‐differences estimate, 46 prescriptions per 1000 men, *p* < 0.001).

**Conclusions:**

Adoption of in‐office dispensing by independent urology groups increased oral specialty drug prescriptions for advanced prostate cancer within a group's market.

## 1 Introduction

Declining physician reimbursement and rising administrative costs have prompted some clinicians to expand service lines to provide additional revenue to support their group practice [1, 2, 3]. In the context of prostate cancer, service line expansion (i.e., providing clinical services onsite that were previously provided elsewhere) by independent urology groups has included radiation therapy, diagnostic laboratory and anatomic pathology services, and conventional and advanced imaging [4, 5, 6], among others. With a paradigm shift in the treatment of advanced prostate cancer to the use of oral specialty drugs [7, 8], independent urology groups are increasingly providing these medications directly to men by dispensing them in the office through specialty pharmacies [9, 10, 11].

The implications of this emerging delivery model are poorly understood. Importantly, the phenotype of urology groups adopting in‐office dispensing has not been characterized. Dispensing of drugs at the point of care is convenient for patients and may improve access and adherence to recommended therapy, particularly for socioeconomically disadvantaged populations at risk for disparities [12]. Oral specialty drugs for prostate cancer are expensive and may be acquired at a discount from group purchasing organizations. Because the cumulative cost can exceed tens of thousands of dollars per patient annually [13, 14], urology groups have the potential to generate a margin, either through discounts themselves or through rebates provided by the pharmaceutical industry [15]. While financial incentives in prostate cancer have spurred utilization and overtreatment in other contexts [5, 16], their effects on use of oral specialty drugs are uncertain.

A retrospective study using national Medicare data was performed to characterize the phenotype of independent urology groups adopting in‐office dispensing and its effect on utilization of oral specialty drugs. Understanding these issues is important as pervasive workforce shortages across most specialties, with associated long wait times for new patient appointments, may limit access to this important class of drugs [17].

## 2 Methods

A retrospective cohort study was performed using a 20% national sample of men with advanced prostate cancer enrolled in Traditional Medicare and managed by independent urology groups between 2011 and 2019. Advanced prostate cancer was defined using an established algorithm based on the initiation of chronic androgen deprivation therapy (bilateral orchiectomy or continuous treatment of at least 6 months with leuprolide, goserelin, degarelix, or triptorelin) [18, 19]. Men receiving androgen deprivation therapy as an adjunct to local therapy were excluded. The study was limited to men with advanced prostate cancer managed by urologists in independent medium (3–9 urologists) and large (≥ 10 urologists) urology groups based on prior work suggesting that in‐office dispensing was largely limited to this context [10]. Men were assigned to their primary urologist based on the frequency and intensity of evaluation and management visits for each 12‐month period [5, 20]. Urologists were assigned to their respective urology groups using data from the Medicare Data on Provider Practice and Specialty file [21].

The outcome for the study was adoption of in‐office dispensing measured at the urology group level. In‐office dispensing was established annually using data from the National Council for Prescription Drug Programs [22]. Independent urology groups not adopting in‐office dispensing throughout the study period served as controls (i.e., non‐dispensing groups). To understand the phenotype of groups adopting in‐office dispensing, characteristics were measured annually for each independent urology group, including size (i.e., number of urologists), number of men with advanced prostate cancer managed, % of Black men with advanced prostate cancer in a group's patient panel, % of men from a rural zip with advanced prostate cancer in a group's patient panel, and the median age of the group's panel of men with advanced prostate cancer. As a measure of a group's appetite for service line expansion in the prostate cancer context, we characterized whether a group had ownership of a radiation vault, identified using an established and validated algorithm; sensitivity and specificity of 86.8% and 93.6%, respectively [23]. Additional urology group characteristics were measured at the market level. Urology group markets were defined using a variable market area approach, as previously described [24, 25]. This approach is based on the flow of all patients, independent of diagnosis or gender, to a urology group annually. The market area was defined by the shortest distance between the zip code centroid of the urology group and patient from which 75% of all evaluation and management claims were derived in that year. Urology group market variables included competition, assessed using the Herfindahl–Hirschman Index (HHI) [25] and socioeconomic vulnerability. The last was measured using the social vulnerability index (SVI) [26]. The SVI ranges from 0 to 1, with 0 representing the least vulnerable and 1 representing the most vulnerable areas.

As a secondary outcome, use of oral specialty drug use per population was measured before and after adoption of in‐office dispensing relative to control markets (i.e., those urology group markets without adoption). For this urology group‐level measure, the numerator consisted of a count of the number of oral specialty drug prescriptions (i.e., abiraterone, enzalutamide, apalutamide, darolutamide) for advanced prostate cancer prescribed by urologists or advanced practice providers in a group annually using Medicare Part D data from January 1, 2011, through December 31, 2019. The denominator was comprised of the number of male Medicare Part D beneficiaries within the urology group's market annually. To facilitate a differences‐in‐differences framework, non‐dispensing groups were matched to the dispensing groups proportionately based on the year of dispensing adoption. For instance, of urology practices adopting in‐office dispensing, 24% did so in 2015. Therefore, 24% of the non‐dispensing groups were randomly selected and matched as controls for that year.

Finally, regional characteristics that may confound adoption of in‐office dispensing were assessed at the zip code level of the group, including the supply of urologists per population, the supply of radiation oncologists per population, hospital beds per population, and the percentage of Medicare enrollees participating in Part C (i.e., managed care penetration).

## 3 Statistical Analysis

Group‐level and regional characteristics were compared between dispensing and non‐dispensing urology groups in the year prior to adoption of in‐office dispensing (or the corresponding year for matched non‐dispensing urology groups). Statistical inference was made using Pearson's Chi‐squared test.

Multivariable logistic regression model was used to assess the relationship between urology group‐level characteristics and adoption of in‐office dispensing by a group, adjusting for regional confounders. Only group‐level and regional characteristics with a *p*‐value < 0.05 on bivariate analyzes were included. Model discrimination was estimated using the area under the curve (AUC).

**FIGURE 1:**
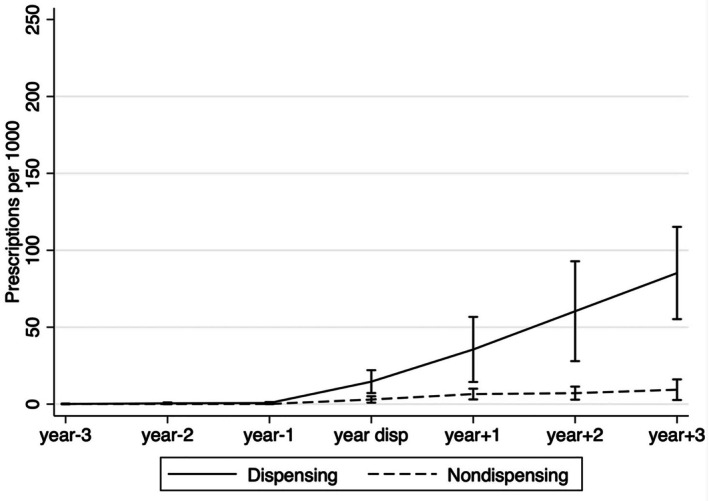
Trends in prescriptions for oral specialty drugs for advanced prostate cancer per 1000 men in adopting and non‐adopting urology groups markets, matched on pre‐period prescribing trends (*n* = 181). The statistical test in the pre‐period for the two slopes (*p* = 0.8) supports that the parallel trends assumption was met.

To estimate the effect of adoption of in‐office dispensing on prescriptions per population, a multivariable linear regression model was used, with standard errors clustered at the urology group‐level. To facilitate the difference‐in‐differences design, an interaction term between dispensing (yes/no) and time (pre/post dispensing) was included. Because trends in prescriptions per population in the pre‐dispensing period were not parallel between adopters and non‐adopters (see Figure S1, *p* < 0.01), it was necessary to further match urology groups based on prescriptions per population in the “pre” period to fulfill criteria for the differences‐in‐difference design [27]. This resulted in a subset of 181 groups (57 adopting in‐office dispensing and 124 controls) for the analysis. In this subset, parallel trends were confirmed by visual inspection (Figure 1) and statistical inference (interaction test for pre‐period time trend *p* = 0.8). The multivariable linear regression model was adjusted for practice size, advanced prostate cancer panel size, % Black race, % rural residence, radiation vault ownership, group competition, SVI of a group's patient panel, regional hospital bed supply, and regional managed care penetration.

All analyzes were carried out using Stata 17 (College Station, TX). All tests were two‐sided with the probability of type 1 error (α) set at 0.05. The study protocol was judged to be exempt from review by the institutional review board.

## 4 Results

**FIGURE 2:**
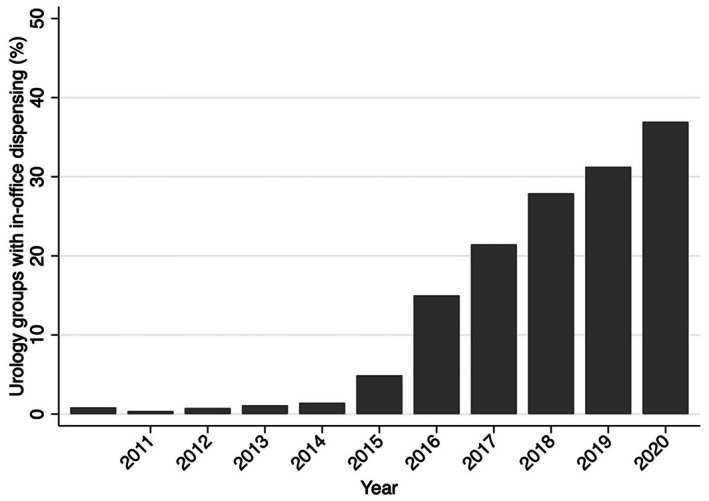
The percentage of medium and large independent urology groups with in‐office dispensing by year. Between 2011 and 2019, in‐office dispensing increased from 0.4% to 37% (*p*‐value for trend < 0.001).

Of 276 independent urology groups, 117 adopted in‐office dispensing and 159 did not. The percentage of urology practices that opened a dispensing pharmacy between 2011 and 2019 increased substantially from 0.4% to 37% (*p*‐value for trend < 0.001; Figure 2). There were notable differences between adopting and non‐adopting urology groups (Table 1). Those adopting in‐office dispensing were larger in size and more commonly owned radiation vaults. Adopting groups also managed more men with advanced prostate cancer. Finally, adopting groups were more likely to care for patients in urban and less socially vulnerable areas.

**TABLE 1:** Characteristics of adopting and non‐adopting urology groups in the year before in‐office dispensing.

	Dispensing	Non‐dispensing	*p*
Number of practices	117	159	
Advanced prostate cancer patients (Median, IQR)	90 (45–160)	30 (15–60)	< 0.001
Age, Median (IQR)	79 (77–80)	79 (77–81)	0.98
% patients of Black race, Median, (IQR)	9.3 (0–18)	1.2 (0–13)	0.14
% Rural location, Median (IQR)	14 (10–16)	36 (31–41)	0.021
Social vulnerability, Median (IQR)	0.43 (0.34–0.61)	0.55 (0.39–0.68)	< 0.01
Herfindahl–Hirschman Index, Median (IQR)	4819 (3881–6800)	4608 (3251–6647)	0.09
Urologists per 100,000 residents			0.38
Low (≤ 44)	36 (31)	55 (35)	
Medium	45 (38)	49 (30)	
High (≥ 68)	36 (31)	55 (35)	
Radiation Oncologists per 100,000 residents			0.49
Low (≤ 19)	37 (32)	58 (37)	
Medium	40 (34)	57 (35)	
High (≥ 29)	40 (34)	44 (28)	
Hospital beds per 100,000 residents			0.28
Low (≤ 4580)	35 (30)	62 (39)	
Medium	40 (34)	48 (30)	
High (≥ 6340)	42 (36)	49 (31)	
Medicare Managed Care penetration			0.06
Low (≤ 15%)	30 (26)	62 (39)	
Medium	42 (36)	46 (29)	
High (≥ 28%)	45 (38)	51 (32)	
Large practice, ≥ 10 physicians (%)	58 (50%)	20 (13)	< 0.001
Radiation vault ownership (%)	40 (34)	10 (6)	< 0.001

As illustrated in Table 2, there were several urology group characteristics associated with adoption of in‐office dispensing in the year prior to adoption, including large practice size (OR 2.9, 95% CI 1.2–6.5), advanced prostate cancer panel size (OR 1.1 for each 10‐patient increase, 95% CI 1.0–1.2), lower socioeconomic vulnerability (OR 0.81 per 0.1 unit increase in the SVI, 95% CI 0.69–0.95), less urology group competition (OR 1.02 per 100 unit increase in the HHI, 95% CI 1.0–1.1), and radiation vault ownership (OR 3.1, 95% CI 1.1–8.4). The AUC for this model was 0.83 (Figure S2).

**TABLE 2:** Multivariable logistic regression between urology group characteristics and adoption of in‐office dispensing.

	Odds ratio (95% CI)	*p*
Practice size		
Medium (3–9)	Referent	
Large (≥ 10)	2.8 (1.2–6.5)	0.015
Number of advanced prostate cancer patients (per 10 patient increase)	1.1 (1.0–1.2)	0.018
% Black men in the panel of advanced prostate cancer patients		
1 (< 1%)	Referent	
2 (1%–11%)	1.5 (0.61–3.7)	0.38
3 (> 12%)	2.0 (0.94–4.4)	0.072
Social vulnerability (per 0.1 increase in the SVI)	0.81 (0.69–0.95)	0.009
Urology group competition (per 100 unit increase in the HHI)	1.02 (1.0–1.1)	0.004
Place of residence		
Rural	Referent	
Urban	2.4 (0.97–5.9)	0.060
Medicare Advantage Penetration		
Low (≤ 15%)	Referent	
Medium	2.0 (0.9–4.3)	0.087
High (≥ 28%)	2.7 (1.2–6.2)	0.016
Radiation vault ownership		
No	Referent	
Yes	3.1 (1.1–8.4)	0.027

**FIGURE 3:**
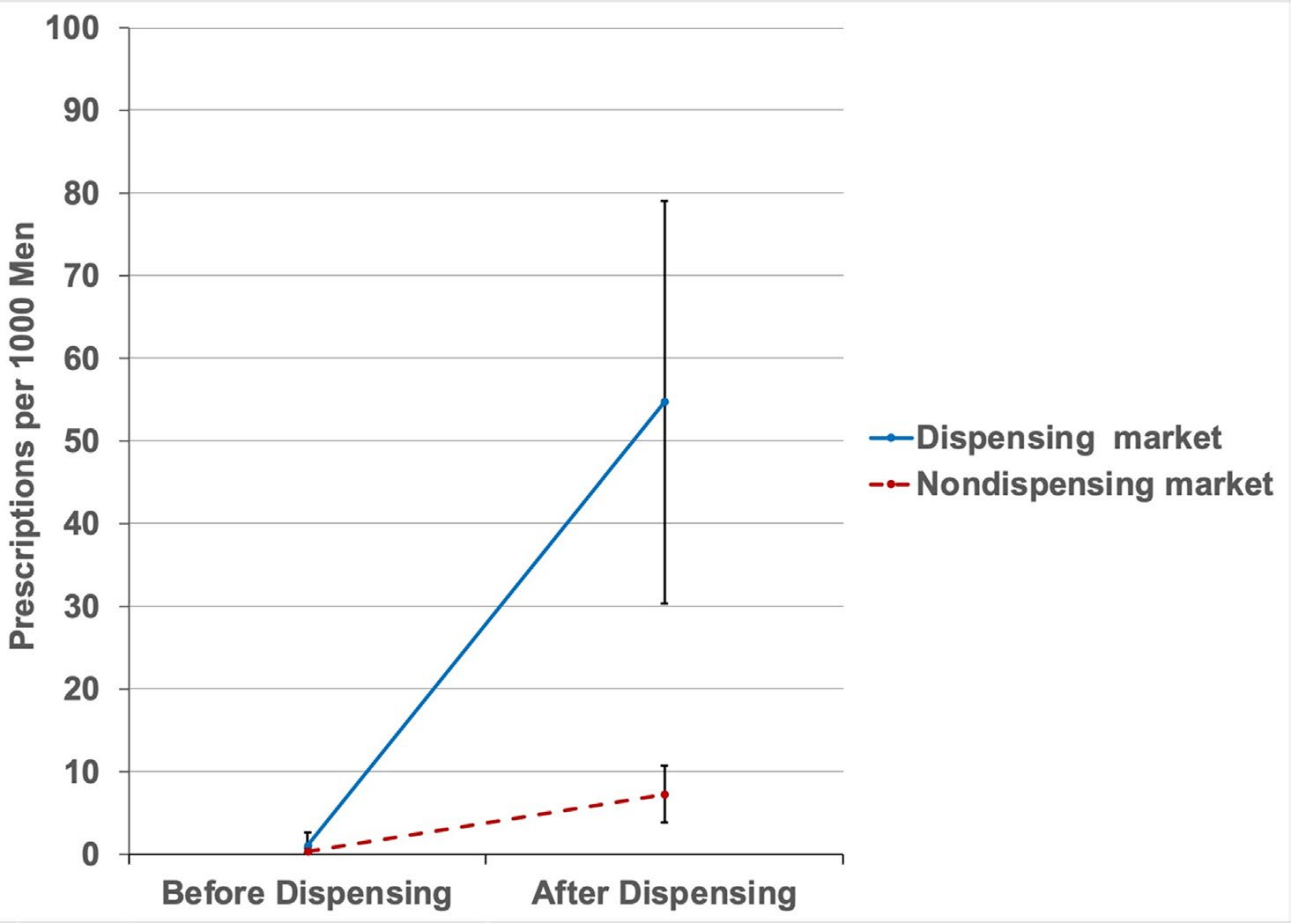
Oral specialty drug prescriptions per 1000 men in an adopting and non‐adopting urology group market, before and after adoption of in‐office dispensing. Adjusted difference‐in‐differences estimate, +46 prescriptions per 1000 men (*p* < 0.001) in adopters relative to non‐adopters.

As shown in Figure 3, adoption of in‐office dispensing was associated with a significant increase in prescriptions per population in a urology group's market relative to those markets without adoption (adjusted difference‐in‐differences estimate, +46 prescriptions per 1000 male Medicare beneficiaries, *p* < 0.001).

## 5 Discussion

Independent urology groups adopting in‐office dispensing have a phenotype favorable to service line expansion, including larger practice size to defray capital outlays, a less competitive marketplace to better capture unmet clinical demand, an ample panel of men with advanced prostate cancer who may benefit from treatment, and a milieu typified by a less socioeconomically vulnerable population that can afford the hefty out‐of‐pocket costs associated with these therapies. Further, adopters were more likely to have invested in a radiation vault, supporting their penchant for service line expansion into non‐traditional, but related, areas of clinical practice. Finally, adoption of in‐office dispensing by independent urology groups was associated with a significant increase in oral specialty drug use within their markets relative to non‐adopters.

Urology groups increasingly have adopted in‐office dispensing over the past decade [9]. Proponents of this delivery model have pointed to the convenience it affords patients. They argue that in‐office dispensing may improve compliance with recommended therapy, the potential to enhance access to this important class of medications, and foster continuity of the patient‐physician relationship [28]. Notably, evidence suggests that point‐of‐sale prices for expensive medications, such as oral specialty drugs for advanced prostate, are significantly less costly when dispensed in the office, relative to retail or specialty pharmacies [9]. Finally, as adopting in‐office dispensing requires substantial financial commitment to navigate regulatory requirements, secure the necessary human capital, and acquire the expensive medications, adopting groups would be highly motivated to help patients navigate the considerable economic barriers (e.g., through enrollment in assistance programs) to start and remain on treatment [29].

Nonetheless, one potential pitfall of this emerging delivery model relates to the financial incentives at the level of the urology group. Costs for oral specialty drugs for prostate cancer approach $10,000 per month of treatment [13, 14]. Urology groups with in‐office dispensing may benefit directly from prescribing these medications through rebates from their group purchasing organization or through margins on reimbursement from the drug itself [9, 30]. Even small margins on these expensive drugs have the potential to contribute considerably to the net revenue of the urology group, making incentives for utilization strong. There are several reasons to suspect that financial incentives in healthcare may play a role in physician behavior. Prior work suggests that medical oncologists preferentially use regimens comprised of high‐margin drugs in scenarios in which equally effective and less expensive alternatives are available [31]. In the context of urology groups that manage prostate cancer, urology group ownership of radiation vaults was associated with higher rates of treatment in men unlikely to benefit [5]. Further, subsequent divestment of vault ownership lowered rates of treatment with radiation in unhealthy patients to a level similar to that of non‐owners [16]. With oral specialty drugs for advanced prostate cancer, adoption of in‐office dispensing led to a significant increase in prescriptions within the group's market. However, it is unclear to what extent financial incentives affect high‐risk or inappropriate prescribing (e.g., treatment in men unlikely to benefit, those at higher risk for adverse events, inappropriate combinations with other therapies [32]). This is particularly relevant in the modern era, where these drugs increasingly are prescribed for longer periods of time as indications broaden to more proximal stages of the disease [33, 34].

This study should be interpreted in the context of its limitations. First, this study included independent urology groups comprised of 3 or more urologists, which limits the generalizability of the findings to these groups. However, prior work suggests that these groups are the most likely to engage in service line expansion [5]. Second, this study did not assess the appropriateness of oral specialty drug use for advanced prostate cancer. Thus, while adoption of in‐office dispensing led to a significant increase in use of these drugs, this is not necessarily a bad thing. Indeed, improving access to and compliance with this important class of life‐prolonging drugs may have a positive effect on population health. Next, while we matched dispensing and non‐dispensing practices based on year and the number of oral specialty drug prescriptions, practices in the two groups still varied by a few baseline characteristics. However, we were able to adjust for notable differences in the multivariable models, in an attempt to reduce bias, though it is possible that unmeasured confounding remains given the study design.

This study has implications for policymakers who are naturally interested in the effects of physician practice organization, financial incentives and service line expansion. There is growing data on the interplay between these factors in the context of reduced physician reimbursement rates and rising administrative costs, prompting practices to align themselves with models that are financially beneficial. Reorganization of the physician workforce, through integration with other groups, hospitals or by acquisition of for‐profit private equity firms [1, 35], may facilitate entrepreneurial activities that require large capital outlays, such as radiation vaults, ambulatory surgery centers, and advanced imaging facilities. While the benefits of expanding service lines may include increased access and convenience for patients, potential financial incentives inherent in these relationships may influence physician behavior, as previously demonstrated [5]. More recently, the acquisition of independent urology by private equity firms continues to accelerate with most recent estimates suggesting that there are nearly 5800 private equity physician practice sites in 2021 [36]. Firms acquire practices with potential for growth in the short term (i.e., 3–7 years) and make decisions to increase the value of the group [37], such as cutting administrative costs, increasing practice throughput, and expanding service lines. While some suggest that these decisions adversely affect patient care [38], the data is mixed. For instance, some have demonstrated that acquisitions have led to increased mortality among nursing home residents [39] and increased hospital‐acquired adverse events [40]. However, others have shown that these acquisitions can lead to improved outcomes among patients hospitalized for certain conditions [41]. The concerns surrounding these acquisitions parallel those related to the dispensing pharmacy delivery model, where the financial incentives for the group (i.e., increased utilization, use of expensive therapies) may not necessarily align with quality care. Thus, stakeholders must ensure that quality is preserved, costs are controlled, and access to treatments is maintained. This is particularly relevant as more patients are expected to be candidates for second generation oral anti‐androgens, as the indications for these therapies shift to less advanced disease [8]. Furthermore, given that decisions in advanced prostate cancer often involve collaboration between multiple specialties, particularly medical oncologists, it will be important to understand the collaboration between urologists and medical oncologists in the context of dispensing pharmacies.

## 6 Conclusions

Independent urology groups that adopt in‐office dispensing have a phenotype that is favorable to service line expansion, including a larger panel size of men with advanced prostate cancer who are more likely to reside in urban and less socioeconomically vulnerable markets. Further, adopting groups face less competition within their markets and are more likely to have invested in radiation vaults, supporting an appetite to expand into new areas of treatment. Adoption of in‐office dispensing was associated with a significant increase in prescriptions for oral specialty drugs for prostate cancer within urology group markets. Future work should explore the effect of in‐office dispensing by urology groups on the quality of care in men with advanced prostate cancer.

## Author Contributions

**Kassem S. Faraj:** conceptualization‐equal, formal analysis‐equal, investigation‐equal, methodology‐equal, supervision‐equal, writing – original draft‐equal, writing – review and editing‐equal. **Mary Oerline:** data curation‐equal, formal analysis‐equal, software‐equal, writing – review and editing‐equal. **Samuel R. Kaufman:** data curation‐equal, formal analysis‐equal, resources‐equal, software‐equal, writing – review and editing‐equal.

**Xiu Liu:** data curation‐equal, formal analysis‐equal, resources‐equal, software‐equal, writing – review and editing‐equal. **Christopher Dall:** investigation‐equal, validation‐equal, writing – review and editing‐equal. **Arnav Srivastava:** investigation‐equal, validation‐equal, writing – review and editing‐equal. **Megan E. V. Caram:** conceptualization‐equal, investigation‐equal, supervision‐equal, writing – review and editing‐equal. **Vahakn B. Shahinian:** conceptualization‐equal, funding acquisition‐equal, methodology‐equal, project administration‐equal, supervision‐equal, validation‐equal, writing – review and editing‐equal. **Brent K. Hollenbeck:** conceptualization‐equal, funding acquisition‐equal, investigation‐equal, project administration‐equal, resources‐equal, supervision‐equal, visualization‐equal, writing – review and editing‐equal.

## Funding

Dr. Faraj is supported by the NCI (T32 CA180984). This study was supported by funding from the NCI (R01 CA275993 and R01 CA279746).

## Disclosure

**Precis:** Independent urology groups that adopt in‐office dispensing have a phenotype that is favorable to service line expansion. Adoption of in‐office dispensing was associated with a significant increase in prescriptions for oral specialty drugs for prostate cancer within urology group markets.

## Conflicts of Interest

The authors declare no conflicts of interest.

## Supporting information


**Figure S1:** Trends in prescriptions for oral specialty drugs for advanced prostate cancer per 1000 men in all adopting and non‐adopting urology groups markets (*n* = 276).
**Figure S2:** AUC curve of model predicting adoption of in‐office dispensing, including practice size, advanced prostate cancer panel size, % Black men in advanced prostate cancer panel size, social vulnerability, urban/rural residence, radiation vault ownership, urology group competition and Medicare Advantage Penetration.

## Data Availability

This study used Medicare claims data, provided by the Centers for Medicare & Medicaid Services (CMS) under license/by permission.
