# Supplementary material for: Impact of In‐Office Dispensing Adoption by Urology Practices on Oral Specialty Drug Use in Advanced Prostate Cancer
Source: Cancer Med. 2026 Jan 11;15(1):e71475. doi: 10.1002/cam4.71475 (PMC12791029; doi:10.1002/cam4.71475)

**Supplemental Figure 1.** Trends in prescriptions for oral specialty drugs for advanced prostate cancer per 1000 men in all adopting and non-adopting urology groups markets (n=276).

**Supplemental Figure 2.** AUC curve of model predicting adoption of in-office dispensing, including practice size, advanced prostate cancer panel size, % Black men in advanced prostate cancer panel size, social vulnerability, urban/rural residence, radiation vault ownership, urology group competition and Medicare Advantage Penetration.


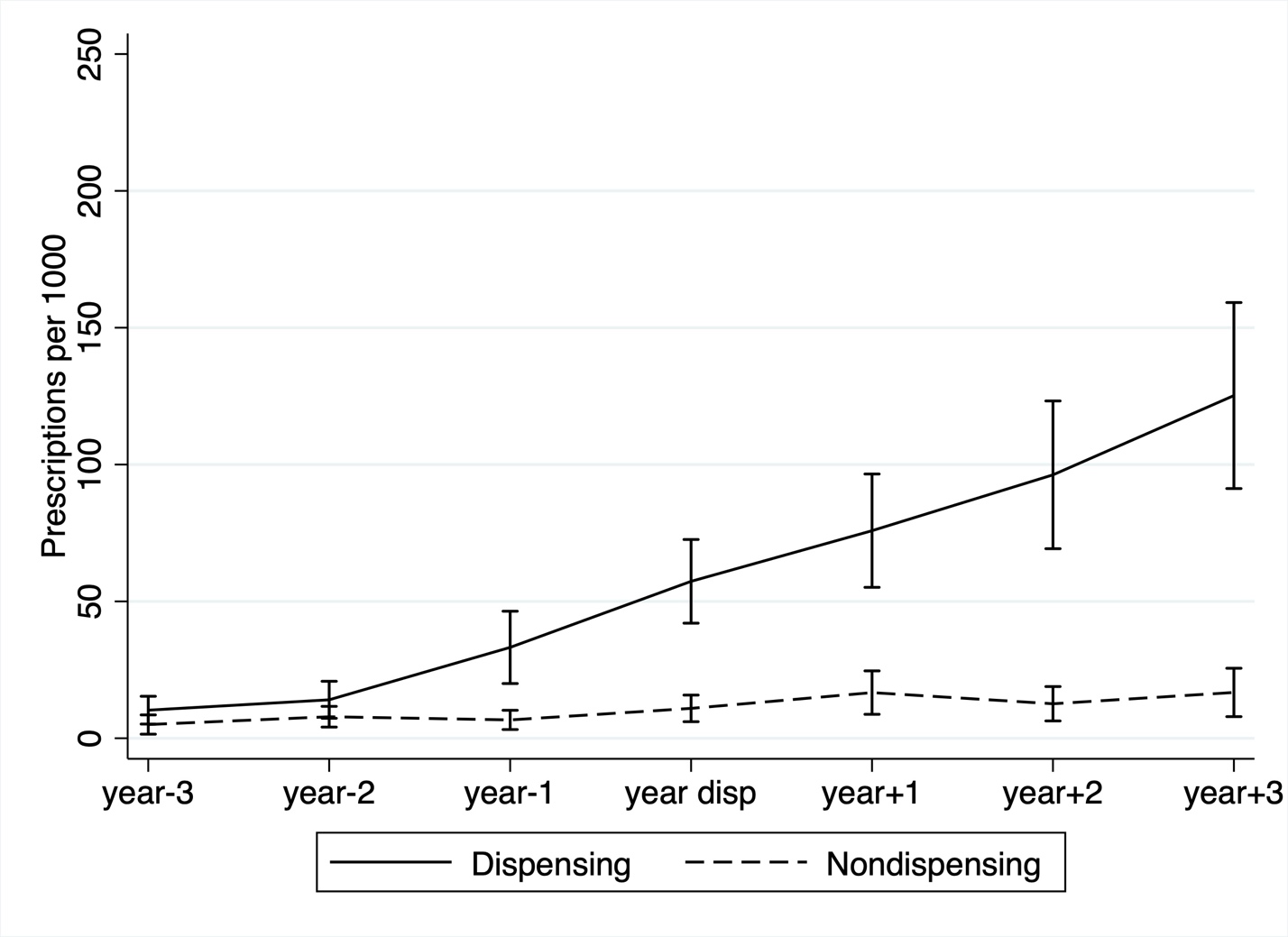
Supplemental Figure 1.

Supplemental Figure 2.


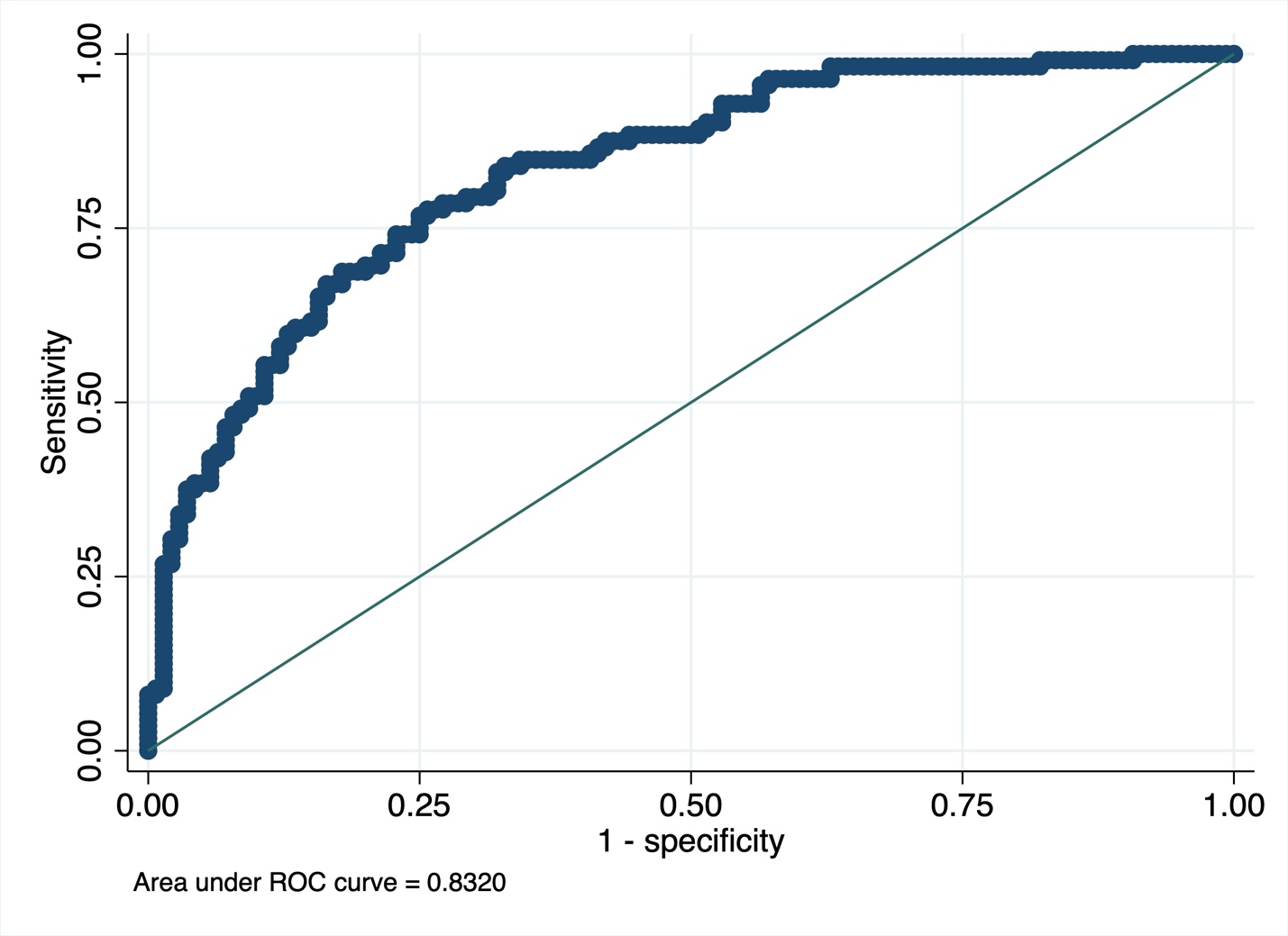

Supplement: Supplementary file 1 — Figure S1: Trends in prescriptions for oral specialty drugs for advanced prostate cancer per 1000 men in all adopting and non‐adopting urology groups markets (n = 276). Figure S2: AUC curve of model predicting adoption of in‐office dispensing, including practice size, advanced prostate cancer panel size, % Black men in advanced prostate cancer panel size, social vulnerability, urban/rural residence, radiation vault ownership, urology group competition and Medicare Advantage Penetration. [file CAM4-15-e71475-s001.docx]
